# Role of social innovations in health in the prevention and control of infectious diseases: a scoping review

**DOI:** 10.1186/s40249-024-01253-w

**Published:** 2024-11-20

**Authors:** Maryam Khazaee-Pool, Tahereh Pashaei, Maryam Zarghani, Koen Ponnet

**Affiliations:** 1 Department of Health Education and Promotion, School of Health, Health Sciences Research Center, Mazandaran University of Medical Sciences, Sari, Iran; 2 Substance Abuse Prevention Research Center, Research Institute for Health, Kermanshah University of Medical Sciences, Kermanshah, Iran; 3 Document Center and Central Library, Medical Information Management, Hamadan University of Medical Sciences, Hamadan, Iran; 4 Department of Communication Sciences, Imec-Mict-Ghent University, Ghent, Belgium

**Keywords:** Social innovation, Infectious disease, Prevention, Control, COVID-19

## Abstract

**Background:**

The implementation of social innovations for addressing societal challenges, particularly in health, leverages community participation and technology to optimally meet social needs compared to traditional approaches. A key feature of these innovations is their ability to utilize existing capacities for contributing to resolving infectious disease outbreaks, which has attracted significant attention from health organizations. Given the potential of these innovations, this study has investigated social innovations in the prevention and control of infectious diseases as one of the major global challenges in the form of a comprehensive literature review.

**Methods:**

This review study examined the relevant literature from January 1, 2010 to December 31, 2022. Based on inclusion and exclusion criteria, 50 documents were retained and fully examined. The documents were analyzed by applying a thematic analysis, and important content related to the application of social innovations for the prevention and control of pandemic infectious diseases was extracted using a data collection form.

**Results:**

Five major themes concerning social innovation in the prevention and control of epidemic diseases were discerned as follows: new products, novel processes and policies, empowerment, innovative practices and behaviors, and community engagement. New products include technological products for control and management of epidemics, preventive products, diagnostic and therapeutic products. Novel processes and policies are related to reorienting and reorganizing care methods, control and monitoring policies, participatory and creative strategies. Empowerment is focused on enhancing the capabilities of health workers, community leaders, and communities. Innovative practices and behaviors involve technology-based participation and support mechanisms. Community engagement is related to awareness, consultation, community mobilization, and participation in production and support.

**Conclusions:**

During the outbreak of infectious diseases, governments are faced with many challenges, including health, economic and social challenges. To answer these challenges, tools should be used that have the ability to answer the problem from several aspects. Social innovation as an appropriate process in response to health crises has led to new forms of relationships and empowered the communities. And to promote public health, it provides the opportunity for all members of the society to participate in crisis resolution and optimal use of resources.

**Supplementary Information:**

The online version contains supplementary material available at 10.1186/s40249-024-01253-w.

## Background

From the beginning of twentieth century, numerous epidemics have occurred, including Spanish flu of 1918 caused by influenza A (H1N1) virus; 1957 influenza A (H2N2) virus; 1968 influenza A (H3N2) virus; severe acute respiratory syndrome (SARS) epidemic of 2002; 2009 swine flu outbreak as a result of Influenza A virus subtype H1N1 (A/H1N1); Ebola virus disease (EVD) outbreak in West Africa between 2013 and 2016, and most recently, coronavirus disease 2019 (COVID-19). The latter, which emerged in December 2019, has affected almost every country in the world [1]. The COVID-19 pandemic deeply touched all sections of society not just in terms of health effects, so that many countries developed new approaches to control this disease. Outbreaks of diseases such as Zika fever, Middle East respiratory syndrome coronavirus (MERS-CoV), and Ebola have shown that the most effective method of organizing responses to health crises is confidence in people and facilities, considering public perspectives and proactively sharing information with people to keep them safe [2]. Another important way of responding to health crises is social innovation, which refers to new solutions that are more effective than existing ones for meeting social needs and increasing people’s ability to take action when dealing with complex or unsolvable difficulties [3].

COVID-19 has aggravated the already existing deficits of health institutions, including the lack of valid and reliable information regarding the quality and availability of providers, enforcement and recourse mechanisms, as well as quality of services, tests, equipment, and drugs [4]. This situation has opened a window of opportunity for innovations in various areas, including prevention and health promotion strategies as well as diagnostic and therapeutic strategies. In early 2000s, the term "social innovation" that was actively used in the social context was on political agenda of many countries to describe new modes of interaction between authorities in different sectors with people to solve social problems.[5] Social innovation (SI) is a creative recombination of existing assets aimed at achieving well-known social goals for meeting social needs through collaboration [6], believing that community and all members of society are competent interpreters of their own lives and have the capacity to solve their problems [7]. Social innovation can be useful for combining skills that can be applied by societies to achieve what is most valuable in life.[8] SI emphasizes ideas and solutions shaping social value regardless of their origin; SI is an important element of communities’ capacity to adapt because it encourages communities and individuals to actively interpret their lives and participate in creatively solving health challenges [9].

The epidemics of the last decade and COVID-19 have clearly shown how disease outbreaks can spread rapidly in society, which has revealed the gaps in their management. The increase in the burden of these diseases in the field of health care as well as their prevention and control programs are considered a convincing reason to address the gaps in the provision of health services [10]. Therefore, the leading organizations in health and welfare have focused on social innovations, especially based on participation of communities in the prevention and control of diseases, and different frameworks and strategies have been presented in this field and provided to care organizations in the country. WHA Resolution 75.13 was adopted by WHO member states to improve surveillance and follow-up programs at national, subnational and facility levels in accordance with the core components recommended by WHO. The Global Strategy on Infection Prevention and Control (GSIPC) was developed as a global action plan and a regulatory framework in consultation with WHO member states and regional economic integration organizations based on a people-centered approach focusing on the protection of health workers, as well as safety and empathy with the patient to ensure fairness, accountability and sustainability in care related measures [10]. The Social Innovation in Health Initiative (SIHI) led by the United Nations Development Programme (UNDP)/ the Special Programme for Research and Training in Tropical Diseases (TDR)^1^ was launched in 2014 with the aim of identifying, strengthening and developing solutions to local health problems, which was a useful initiative for adopting health measures to promote health [11]. The Outbreak Preparedness Framework means preparedness and response for the prevention and control of communicable diseases that can strengthen the health care environment. In fact, this tool has been designed with a set of key measures to support countries through developing activities or measures for preparing and responding to the outbreaks of infectious diseases [12].

Conceptual framework of relationships and priorities in infection prevention and control contributes to decision-making for health and environmental systems in the direction of sustainable development goals based on three priorities of strengthening the evidence base, measuring Infection Prevention and Control (IPC), reducing environmental impacts and using the power of human behavior in IPC programs in accordance with evidence-based information; this tool is people-oriented and adheres to the principles of sustainable development [13]. The models using social innovation in health accelerate a participation platform of SIH centers and an interdisciplinary, intersectoral infrastructure for the adoption of social innovation programs. The Riders for Health project, which started in Lesotho and expanded to Liberia, Kenya, Zimbabwe, Zambia, Malawi, Gambia and Nigeria, has proposed a three-stage social participation process for social innovation in health [14]. Indeed, prevention and control measures are beyond a specialized field, and the success of a program depends on behaviors of individuals within the system who should adhere to a social science approach focusing on community health to achieve an enabling environment for IPC programs that are evidence-based and follow the principles of sustainability [13].

With respect to public health projects, the goal of social innovation is to improve the sustainability and resilience of communities. Therefore, the Social Health Innovation Initiative was launched by Tropical Disease Research program in 2014 to promote social innovation for communities affected by infectious diseases with the goal of achieving sustainable development [9]. Innovation in care models has brought about positive results, including increased access to health services, improved affordability, and enhanced effectiveness of indicators of disease or well-being [8].

The world is now confronted with unprecedented social and economic challenges in the field of health care due to COVID-19 pandemic, and governments throughout the world have introduced policies and procedures to address the spread of this virus. These measures range from counseling communities and social distancing to more draconian measures such as blockage of borders [10]. It is important to consider how social innovation can be effective in controlling and managing epidemics and public health crises. Therefore, the purpose of this article is to review the literature related to the use of social innovations in the management and control of epidemic diseases. Based on the global experience published in various studies, our goal is to determine what aspects of these innovations have been used in the case of increasing spread of COVID-19 and other epidemics of the past years.

## Methods

This research is of the scoping review type, which adopts a qualitative approach and thematic analysis to identify themes from research texts related to the application of social innovations in the control and prevention of epidemic infectious diseases. Thematic analysis is a qualitative research method utilized to identify themes, i.e. patterns present in the important or interesting data of the researcher to answer the research questions or provide information about the problem under investigation by the researcher [16, 17]. The method is not just summarizing the information from the investigated texts, but rather understanding and interpreting them according to the purpose of the research.

### Search strategy

The search for articles was done in international electronic databases (PubMed, Scopus, and Web of Science), and a snowball search method was used to access published reports related to social innovation. Keywords were selected based on the subject of study as well as terms extracted from the medical thesaurus (MeSH) on PubMed portal. The search was performed based on the keyword combinations shown in Additional file 1. The search was limited to title, abstract, and keyword fields and to papers published between January 1, 2010, and December 31, 2022. The following keywords were used: ("Social Innovation" OR "health innovation" OR "Community Engagement" OR "Community Participation*") AND ("COVID-19" OR "SARS-CoV-2" OR "SARS CoV 2 Virus" OR "COVID-19 Virus" OR "Novel Coronavirus" OR "Coronavirus" OR "Communicable Disease Control*" OR "Communicable Disease").

### Selection and evaluation process

In the next step, the results retrieved from each database were transferred to the Endnote X9 resource management software (Clarivate, USA). After removal of duplicates and initial evaluation of results, the title, abstract, and keywords of each study were reviewed based on the inclusion and exclusion criteria outlined below. After confirming the correspondence of documents to the criteria and before reading the full texts, the articles were checked against Web of Science, Scopus, and PubMed databases as well as websites of individual journals to ensure that none of the papers had been retracted. Other sources were assessed by the research team based on organizational credibility and content review.

### Inclusion criteria

The following inclusion criteria were used: (a) studies describing an aspect of social innovation in a health crisis; (b) research focusing on controlling or preventing COVID-19 or other infectious diseases (e.g., Ebola, SARS, Middle East respiratory syndrome, Zika fever); and (c) studies published over 2010–2020 period.

### Exclusion criteria

The exclusion criteria were as follows: (a) studies not related to the objectives of this project; (b) research not focusing on prevention or control of infectious diseases; (c) studies published before 2010; (d) research that was part of a book, letter to editor, abstract, or comment; (e) studies for which the full texts were not available; and (f) documents in which the study population, sample, and research method had not been mentioned.

### Study selection, data extraction, and analysis process

The researchers (MK, TP, and MZ) analyzed the search results to find potentially eligible studies. The approved documents were then examined using thematic content analysis and manually analyzed based on the data collection form (Additional file 2). The following data were extracted from the final set of documents: title, first author, year of publication, study method, specific social innovations for preventing and controlling infectious diseases, and results section. Moreover, the researchers individually screened the title, abstract, and keywords of all articles that exactly matched the inclusion and exclusion criteria.

The findings were shared after completing the title and abstract selection. To increase the rigor of the selection process, only documents that were omitted by all three authors during the title and abstract screening were excluded from full-text screening. Additionally, documents without an abstract were included in the full-text screening. The researchers conducted the full text screening individually and shared their findings. Any conflicts were settled through a discussion with the senior author (MK). The selected documents addressed the specific social innovations in the control and prevention of epidemics as well as their application with an emphasis on COVID-19, which were clustered into separate themes. These thematic axes are discussed in detail in the results and discussion sections.

### Quality assessment of the studies

To augment the quality of analysis, each study was critically evaluated by all members of the research team, and the opinions of all of them were considered when extracting the components. Any disagreement during this process was resolved by a fourth reviewer of the research team, which led to the extraction of specific social innovations for the control and prevention of infectious diseases. Also, the quality of the selected articles was assessed using the Joanna Briggs Institute Prevalence Critical Appraisal Tool [11]. Each article was scored using 10 quality control components suggested by this tool, so that one point was considered for each item. Finally, the total scores were categorized based on the following pattern: (7–10) high quality, (4–6) medium quality and (3–0) low quality [12] (Additional file 3).

## Results

**Fig. 1:**
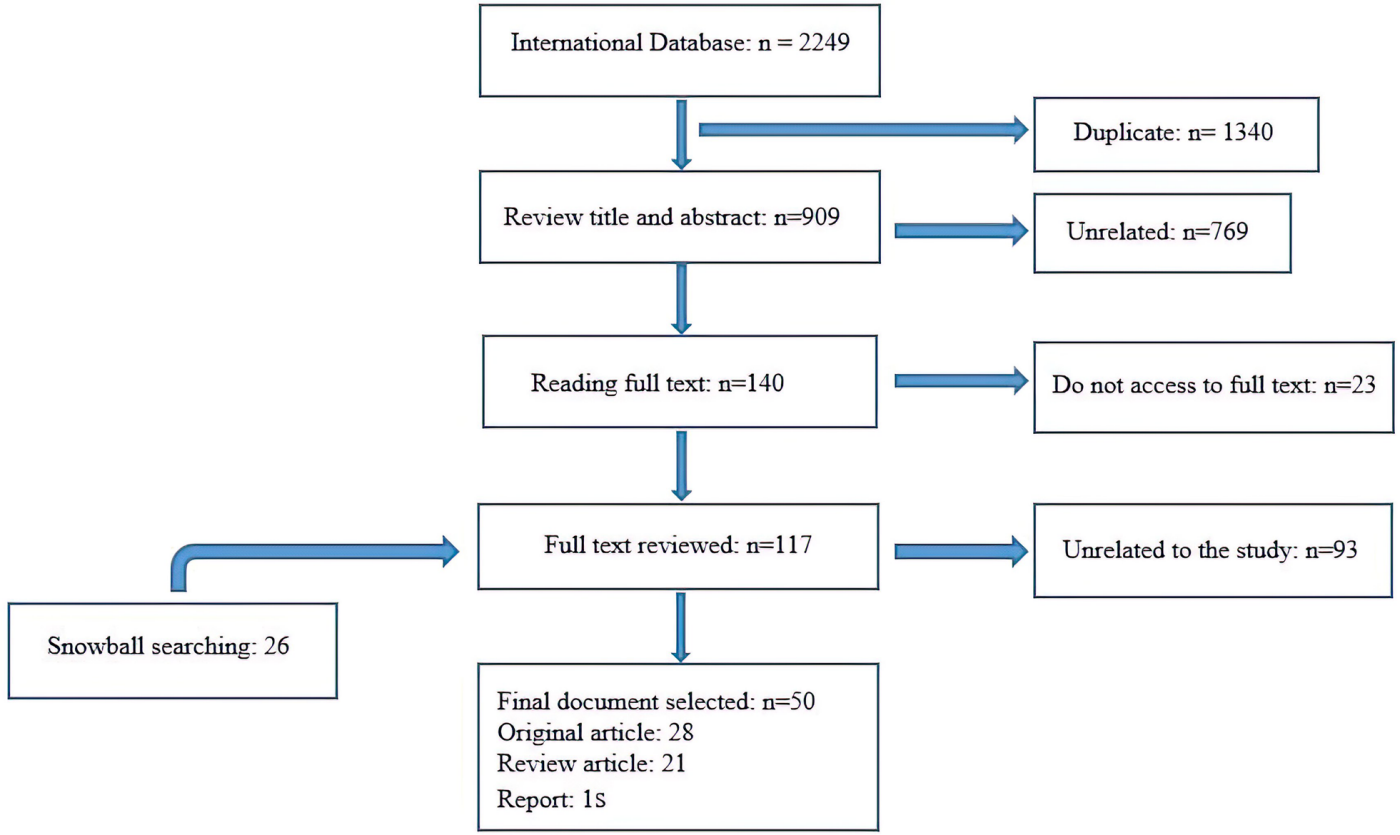
The selection process of the articles

**Fig. 2:**
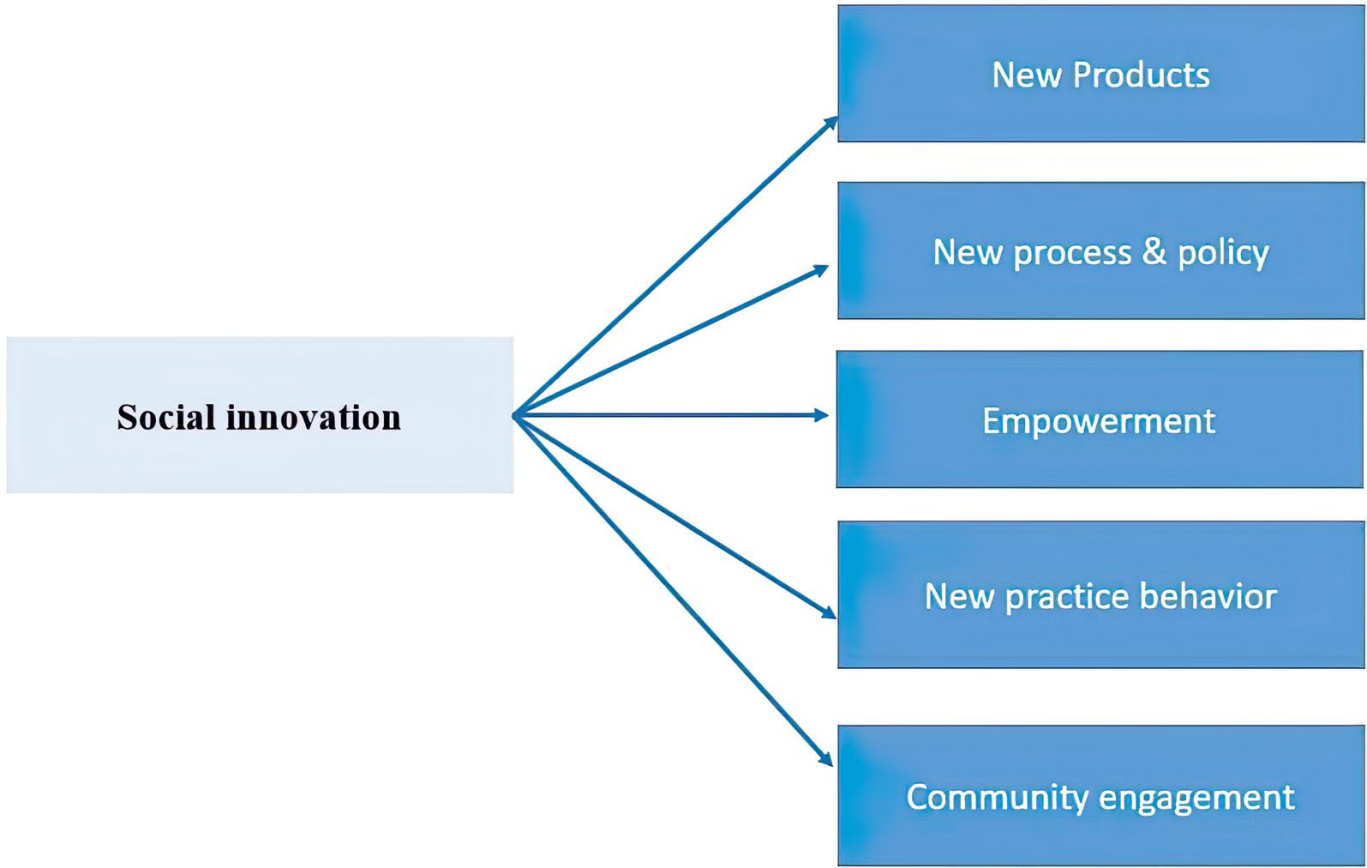
Social innovation themes extracted for the prevention and control of infectious diseases based on literature published in 2010–2022

A total of 2277 records were retrieved. After identifying and deleting 1340 duplicates, 909 records remained for the review of titles, abstracts, and keywords. From screening the title and abstract, it was found that 769 articles were not related to the research topic. Afterward, 140 documents were selected for full-text review. Of these, 23 articles were excluded due to lack of access to the full text, and 117 full-text documents and 26 items of snowball searching were reviewed for the final study. A total of 93 articles were excluded due to their content that was not related to research topic. Finally, relevant data were extracted from 50 documents (Additional file 4). The process of selecting documents is shown in Fig. 1. The extracted themes for social innovation in the prevention and control of epidemic diseases are reported in the following (Fig. 2 and Table 1).

**Table 1:** Role of social innovation in the prevention and control of infectious diseases based on the literature published between 2010–2022

Theme	Subthemes	Components
New products	Technological products for epidemic control and management	Use of digital health tools in emergencies
		Health care a ‘no touch’
		Innovative medical and protective equipment in an epidemic
		New medical facilities
		Digital technology or telehealth services
	Preventive products	Mobile health apps
		Remote primary care clinics
		Remote case identification
		Personal protective equipment
		Product design especially in the for disinfecting purposes and prevention
	Diagnostic-therapeutic products	Point-of-care testing
		Production of respiratory equipment for intensive care units
		Web-based triage tools
		Pharmaceutical innovations in health
		Virtual health care
New processes and policies	Reorienting and reorganizing care pathways	Changes in service delivery
		Creation of medical corps
		Providing mobile units for the delivery of treatment
		Hybrid communities (virtual & face-to-face)
		Home-based health care
		New medical facilities
		Improvement the processes and products
	Control and monitoring policies	Social distancing
		Quarantine
		Contact tracing and movements
		Unity of command in epidemic control
		Enforcement of control rules
	Participatory and creative policies	Democratization of innovation policies
		Interdisciplinary approach in innovations
		Collective decision-making
		Creative strategies for fighting epidemics
		Targeted national initiatives for epidemic control
		Strategic protocols
		Interdepartmental cooperation and interdisciplinary actions
		Adjustments to economic and social conditions in an epidemic
	Technology-based epidemic control and management	Location tracking and contact programs
		Creation of social maps for participation
		Implementation of digital alarm systems
		Digital and contactless assistance for protection
		Digital infrastructure for service delivery
		Technological innovation in health
Empowerment	Empowering health workers	Training workshops
		Reduction of crisis constraints through technology
		Building the resilience of health systems
		Collaborative approaches to health empowerment
	Empowering leaders of different communities	Training of community leaders by medical staff
		Joint conversations between community
		Collaboration between community leaders and health workers on epidemic management
		Resilience leadership
		Teaching crisis participation methods
		Empowering of leaders religious to identify and track epidemics
	Empowering people in the community	Social resilience
		Digital closeness
		Upgrading specialized and health knowledge through technology
		Empowering communities in an epidemic
New practices and behaviors	Awareness and participation based on technology	Use of social media to spread health information
		Information sharing under the supervision of health leaders
		Communication with communities through social media
		Dissemination of health information with new technological tools
	Support mechanisms	Awareness and involvement in crisis
		Social media campaigns for assistance and funding
		Interaction between members of different communities
		Provision of local support online
		Empathy arousal
		Collaboration between social workers and health workers
		Community engagement in financing
Community engagement	Awareness and consultation	Awareness among local and religious communities
		Contribution of religious meetings to the fight against COVID-19
		Promotion of citizen participation
	Mobilization of communities	Voluntary assistance from educators, health professionals, and the general public
		Mobilization of the population to fight the epidemic
		Engagement of universities and other organizations in epidemic control
		Participatory actions and systems
		Involvement of social groups in epidemic management
	Participating in health care	Provide health services under the control of local communities
		Health committees at the local level
		Mobilized to use the capacities of the community
		Promotion of the principles of self-care and contact tracing by community leaders
	Participating in production and support	Human resources in the fields of health and epidemic management
		Partnership between the private and public sectors
		Benevolent financial assistance in epidemic management
		Community support in times of crisis
		Participation in the production of health and protection products

### New products

The first theme of social innovation in the field of health is related to new products, which is divided into the following three sub-themes: technological products for control and management of epidemics, preventive products, diagnostic and therapeutic products. Technological products that have been used for control and management of epidemics include digital health tools for emergencies, touchless healthcare [13], innovative medical and protective equipment for dealing with COVID-19 [14, 15], new medical facilities [16], digital or remote service [17]. Digital health technologies were widely used during the COVID-19 crisis, including telecommunication technologies, mobile health apps, wearables, and online health services. Digital health is provided through virtual health care, telehealth, television interactions between health care providers and citizens, and access to online health information through mobile health apps [18]. Digital tools such as Internet of Things (IOT), biosensors, and artificial intelligence were also utilized to meet the dual goals of social distancing and healthcare in a "touchless" emergency situation [13]. Patient monitoring dashboard [19], ultraviolet light disinfection [10], mobile health programs [18], remote primary clinical care [20], remote case identification, personal protective equipment [21], and new product design in the fields of disinfection and prevention [3, 10, 15], mechanical hand washing systems [22] have all been used to manage health care during infectious disease outbreaks.

Diagnostic and therapeutic products include rapid and decentralized diagnostic tests [20, 23], mobile laboratories [23], respiratory devices for intensive care units [14], web-based triage tools [19, 24], pharmaceutical innovations in health [25], and virtual health care [18]. This innovation has been used through new and emerging technologies from simple software systems to robots and artificial intelligence, which are mostly used for diagnosis of COVID-19 as well as for logistics, transportation or auto-cleaning facilities [14, 26]. In addition, point-of-care decentralized test model [20], the Innovation Accelerator for diagnosis of COVID-19 [4], telemedicine digital platform for remote monitoring of long-term patient care [19] are innovations used for equipment in hospitals such as new types of ventilators for intensive care units that have been developed by various companies or startups [14].

### New processes and policies

New processes and policies based on social innovations have been discussed in various studies, including the reorganization of care methods to set up video interactions between patients and health workers [18], virtual care services [16], intermediary and digital services [16], the creation of medical corps [27], novel organizational forms [22], health care delivery at home [19], the provision of care services by mobile units [19], digitization of services [5], hybrid communities (virtual and face-to-face) [28], process and product improvement [22, 29], home production of health products [30], and innovative health facilities [17, 31]. Health systems around the world have generally utilized three common procedures to quickly improve their structure: creation of novel treatment facilities, changes in users of public locations, and reconfiguration of current medical services to accommodate patients with COVID-19 [17].

Changes in emergency control and surveillance policies for monitoring infectious diseases address issues such as social distancing [3, 13, 16, 32–35], quarantine [28], tracking contacts and movement of people in the community [33, 36, 37], integrated command in epidemic control [29, 38], enforcement of control rules [22] and human–machine cooperation [15]. Manual control of contact tracking is impossible due to COVID-19 pandemic as well as high levels of transmission among asymptomatic persons. Use of a contact-tracking app that reminds people of their close contacts and immediately notifies them of positive cases is more effective in reducing the spread of an epidemic, especially when combined with social distancing.[33]

In addition, creative participatory policies have been developed in the context of epidemics in relation to a variety of topics, including democratization of innovative policies [13], interdisciplinary and innovation approaches [3], collective decision-making [13], creative strategies in the fight against epidemics [38], targeted national initiatives for control of epidemics [22], strategic protocols [19], forming temporary advisory groups for dealing with an epidemic [17], establishment of information and communications technology (ICT)-based cooperation [39], cross-sectoral cooperation and interdisciplinary measures [9], community participation in accordance with guidelines [8, 40], adjustment of economic and social conditions during an epidemic [3] and the formation of temporary COVID-19 consultative teams to guide government decisions [17].

The role of technology in controlling and managing epidemics is the last subtheme identified in this category, which involves video interactions between patients and health workers [18], consideration of tools to control and manage the mobility of community members [36], tracking apps for locations and contacts [37, 41], creation of social maps for participation [42], implementation of digital alert systems [43], as well as digital and contactless assistance for protection [44], digital infrastructure for the provision of services [34], digital interactions [16], and health innovation technology [4, 25]. Also, there are mobile apps that assess and record the vicinity between people via Bluetooth, QR code checkpoints, Global Positioning System, and other devices [41].

### Empowerment

The purpose of health empowerment is to promote community development for changing living conditions and engagement activities [45]. Empowerment is a key element of social innovation in response to epidemics, which has been characterized by building social capital and sustaining connections for communities [45]. Empowering different groups of society for responding to COVID-19 and other pandemics can lead to effective control conditions [29]. This component of social innovation is designed in three subcategories. First, the empowerment of health workers can be examined by the following measures: reducing the limitations of crisis through technology [16], health systems resilience [17, 46], health education approaches in crisis [25], decentralization of tasks to local level [29] and key determinants of health system resilience including governance, as well as finance, intersectoral cooperation, community participation for the provision of health facilities, health workers, medical technologies, products and the institution of public health practices [17].

The empowerment of community leaders involves the training of community leaders by medical staff [40], joint discussions between community leaders and health workers on management of epidemics [40], resilience leadership [19, 46], training in crisis participation methods [25], presentation of guidelines for religious communities [40], and leaders’ ability to identify and track epidemics [9]. Critical events such as COVID-19 pandemic require specific and novel health communication and education strategies, through which public health officials must meet public data requirements [47]. Centers for Disease Control and Prevention (CDC) provide guidance and advice for faith-based organizations on how to educate, prepare, and respond to COVID-19 epidemic [40].

The third category is empowering people in the community, which is perhaps the most effective type of empowerment ensuring the preparedness of society in the face of infectious diseases and epidemics through social resilience [22], participatory methods for empowerment [25], promotion of specialized and health-related knowledge through technology [16], and social awareness [33]. Most infectious diseases, including COVID-19, have a greater potential to spread and cause mortality in vulnerable and poor communities that are in need of empowering.[48] In some countries, multilingual hotlines have been established to ensure widespread availability of COVID-19 data. Knowledge sharing platforms are based on the idea of care as an ethical relationship. These platforms emphasize the relationship between attitudes and moral values, emotional relationships, the responsibility of individuals to participate in society and demonstrate understanding and respect for vulnerable individuals and communities, which provides a basis for community integration[49].

### New practices and behaviors

Responding to an epidemic requires behavioral change, and social innovation can pave the way for the emergence of new behaviors at the time of crisis. This theme of social innovation can be divided into two-subthemes: technology-based participation and support mechanisms. Technology-based participation is related to the role of media in disseminating health information and encouraging solidarity between different groups, which includes the use of social media to publish health information and educate people on health matters [9, 29, 32, 49], application of knowledge-sharing platforms [49], sharing of information under supervision of health leaders [49], updating and dissemination of health information by health workers through the media [48], connecting communities through social media [50], and the dissemination of health information via new technological tools [21, 40]. Due to the spread of COVID-19 through close human contact, digital media, particularly social media, have been the main channels for data distribution [51]. The public page MNResearch Link on Facebook is a credible source of health research information utilized to disseminate research on the spread of infectious diseases and to improve community trust and engagement in health research [50]. OpenStreetPay allows users to make digital donations for facilitating non-contact assistance to homeless neighbors [44].

The second subtheme discussed in the literature related to emergence of new behaviors in epidemic conditions is crisis support mechanisms, which includes social media campaigns regarding aid and economic solutions [3], interaction between members of different communities [16], online local support [14], modulation and preparation of stimulus messages [22], promotion of empathy [47], cooperation between social and health workers [10, 40, 52], public participation in funding [23], apps and websites for accessing health information [15, 18]. Social innovation in supplying innovative equipment and facilities to support consumers, employees, and the public health system often focuses on living and protection needs. One such supporting innovation that was implemented during COVID-19 crisis was the service of hotels to provide their own resources and rooms as separate offices for people who cannot work from home, including Amsterdam-based hotel Zoku that offered this service along with 24-h room delivery service [14]. Another type of crisis support mechanism is to evoke empathy from the community, elicit donations and recruit volunteers to address social needs. For example, in the wake of COVID-19 outbreak, Malaysians used hashtags to recruit volunteers supporting and providing food for the poor and the homeless [22, 23].

### Community engagement

Community participation in epidemics has been examined in relation to four subthemes: awareness, control, care, and production support. The aim of participation is to empower local leaders, parents, families, groups, and the community as a whole. It involves scheduled activities to reach, affect, and engage all sections and units of the community in working toward a common goal. Community participation is a process in which individuals and families take responsibility for community’s health and well-being and also build up the capacity for contributing to the development of community [31]. Community engagement includes awareness-raising and counseling within local and religious communities [40, 45], holding religious meetings to mobilize communities for participating in the fight against COVID-19 [40], promoting individual participation [25, 53], and adapting measures to local contexts [43]. New approaches for contributing to combating infectious diseases involve using native abilities to build capacity for health innovations and ensure their appropriateness and sustainability [30]. The CDC provides guidelines for Healthy Community Partnership (HCP), Department of Spiritual Care and Chaplaincy (DSCC), and Medicine for the Greater Good (MGG) to bring faith-based organizations (like mosques and churches) together to participate in controlling COVID-19 [40].

Mobilization of population in the fight against the epidemic [48], participation of universities and other organizations in control of epidemics [3], participatory measures and systems [3], participation of social groups in control of epidemics [43], and stakeholder participation in epidemic control sessions [9, 54] have been considered actions of community participation. Besides, the participation of people is essential for the effectiveness of measures such as social distance and mask wearing [48]. Some of the countries mobilized networks of community health workers to promote community participation in responding to COVID-19. Their roles range from increasing consciousness via house-to-house visits, assisting with contact tracing, maintaining necessary and basic health facilities, offering essential drugs to patients without COVID-19, following or checking observance to quarantine processes, and evaluating the mental health [17]. NGOs enable the establishment of new institutions for solving problems such as COVID-19 crisis as the most important social innovations in times of crisis [39].

Participation in health care addresses issues such as the volunteer role of educators in the provision of care services [43], provision of health services under supervision of indigenous communities [32], mobilization of capacities of the community [10], and promotion of principles of self-care and contact tracing by community leaders [40, 55]. Interaction between local communities and health systems has been shown to be the key for informing service delivery, decision-making, governance, and meeting the needs of communities before, during, and after crises. Community participation strategies such as partnering with local and native leaders and working with community participants to organize messages and campaigns are critical during public health crises and pandemics [17]. Information and communication technology can play a role in changing the culture of health care and encouraging citizen participation in health. The concept of mobile health, or mHealth, is an example of this technology that effectively provides access to information and enables exchange of information[56].

Contribution to the production of care and protection products has been discussed in relation to human resources in the health industry during epidemics [7], partnerships between the private and public sectors [30], partnerships for mobilizing new resources in times of crisis [39], charitable financial assistance for management of epidemics [23], voluntary networks to combat the epidemic [23, 55], asset-based community development and participation [10], and participation in the production of health products [38].

### Limitations

The main limitation of this review is the omission of a number of sources due to lack of access to full texts during the review process. However, the purpose of this study was to quickly review published literature in the field of social innovation in the control and prevention of infectious diseases. While we made efforts to address this issue by contacting the responsible authors to maximize access to resources, we may have missed some sources. We, however, believe that this limitation is relatively negligible due to the wide range of databases searched like Web of science, Scopus and PubMed.

## Conclusions

Because epidemics pose a great threat to global health, security and socio-economic stability, health care facilities should be strengthened within health centers, communities and across borders. To prevent and contain these diseases, while we need to provide safe, effective and quality health care, investing in the capacity for prevention and control measures at the national level and health care centers can reduce the risk of health care-related transmission and contribute to timely control of the spread of disease. Strengthening the preparedness for responding to these crises for prevention and control will lead to stronger responses, contain the spread of the disease and prevent health systems from exposure to pressure [12]. Social innovation as a collective phenomenon enabling the generation of ideas by individuals for working together to achieve prosperity has been considered with the aim of investing in the participation of communities, especially in epidemic crises. Innovation has responded to medical and health needs at different social, managerial and economic levels with pioneering approaches [14]. Although the focus of current prevention and control program guidelines is on reducing infections in healthcare facilities, many benefits of integrating care programs with community participation during an epidemic disease such as COVID-19 have been reported in prevention and control programs at the community level. Consolidation of partnerships with communities and other health programs, such as immunization, public health and emergency response, can provide and support responses beyond the anticipated health facilities for the preparedness of prevention-control measures in crises [13]. Social innovation in health based on key features such as transformation of social relations and identification of social work through digital connection can have positive effects in providing or supporting preventive and control measures that pursue social development and universal health coverage in different societies, especially in low-income countries, which will eventually lead to the improvement of health and influence on societies at the global level [11].

**Fig. 3:**
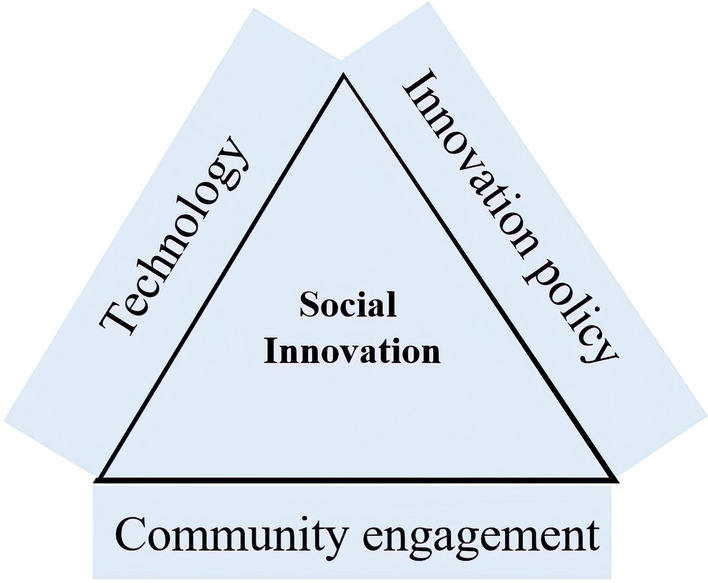
The framework of social innovation in health for prevention and control based on a review of selected studies

Based on the findings, it can be stated that in the crisis of infectious diseases, the use of social innovations in different sectors has been effective for their management. Social innovation provides opportunities for less powerful players (including patients, relations, beneficiaries, community members) to participate in the development of new health solutions [8]. Also, the lessons learned from COVID-19 crisis include a renewed awareness of the need to accelerate innovation and new technology in health care, developing strong community and health system participation and improving data support and decision-making [19]. Effective epidemic control involves diagnosis, reporting, isolation, timely treatment, and social innovation that has proven to be effective in achieving this goal [21]. To expand health innovation, we must also consider social and cultural barriers [30]. The challenges brought about by epidemics are not just medical, methodical, financial, and logistical; they are also social and political. However, epidemics create constraints and lead to loss of resources in terms of product accessibility and time. This means that prioritization may be necessary to resolve and address epidemic challenges [13]. Social innovation enables the mobilization and allocation of resources in society in unusual ways. The technology enabling digital services is an essential tool for advancing such social innovation. In addition, innovation policy-making requires mapping and serious study of social values, preferences, hopes, fears, and power asymmetries that shape the design, development, and implementation of science and technology.[13] This kind of innovation originates from the heart of society and can be promoted by technology and policies. Figure 3 demonstrates the links between these three components in the creation of social innovation. The outputs of social innovation must lower the cost of care, enable the provision of door-to-door services in times of crisis, tackle the problems related with lack of access or sporadic access to care, and include cross-network joint ventures in the health system.

## Supplementary Information

Additional file 1.

Additional file 2.

Additional file 3.

Additional file 4.

## Data Availability

The data collection scales and datasets created and/or analyzed through the present study are available from the corresponding author on reasonable request.

## Footnotes

1 . TDR “is co-sponsored by the United Nations Children’s Fund (UNICEF), the United Nations Development Programme (UNDP), the World Bank and the World Health Organization (WHO)”.

## References

[1] Abayomi A, Balogun MR, Bankole M, Banke-Thomas A, Mutiu B, Olawepo J, et al. From Ebola to COVID-19: emergency preparedness and response plans and actions in Lagos, Nigeria. Glob Health. 2021;17(1):79. 10.1186/s12992-021-00728-x. 10.1186/s12992-021-00728-x PMC8267235 34243790

[2] Al Siyabi H, Al Mukhaini S, Kanaan M, Al Hatmi S, Al Anqoudi Z, Al Kalbani A, et al. Community participation approaches for effective national COVID-19 pandemic preparedness and response: an experience from Oman. Front Public Health. 2020;8:616763. 10.3389/fpubh.2020.616763. 33575243 10.3389/fpubh.2020.616763 PMC7870984

[3] Moura M, Perez IU, Melara LF, Junior JCM. Contemporary design in quarantine: a critical review of design responses to COVID-19 crisis. SDRJ. 2020;13(3):327–41. 10.4013/sdrj.2020.133.03.

[4] Mukherjee K. Integrating technology, innovation and policy: COVID-19 and HTA. Health Policy Technol. 2021;10(1):16–20. 10.1016/j.hlpt.2021.01.003.

[5] Kranzeeva E, Golovatsky E, Orlova A, Nyatina N, Burmakina A. Assessing the effectiveness of social and political innovations in the development of interaction between the authorities and the population during COVID-19: the implication of open innovation. JOItmC. 2021;7(3):172. 10.3390/joitmc7030172.

[6] Prado GC, Leme FDP, Messias LZ, da Costa Miranda NS, de Bona GR. Strategies of design for social innovation and design activism in the promotion of positive social capital during the COVID-19 pandemic in Brazil. SDRJ. 2020;13(3):364–73. 10.4013/sdrj.2020.133.06.

[7] Van Niekerk L, Chater R, Naydenova E, Lim J, Chamas L, Manderson L, et al. Social innovation in health: case studies and lessons learned from low-and middle-income countries. World Health Organization; 2017. https://iris.who.int/handle/10665/259187

[8] van Niekerk L, Manderson L, Balabanova D. The application of social innovation in healthcare: a scoping review. Infect Dis Poverty. 2021;10(1):26. 10.1186/s40249-021-00794-8. 33685487 10.1186/s40249-021-00794-8 PMC7938294

[9] Echaubard P, Thy C, Sokha S, Srun S, Nieto-Sanchez C, Grietens KP, et al. Fostering social innovation and building adaptive capacity for dengue control in Cambodia: a case study. Infect Dis Poverty. 2020;9(1):126. 10.1186/s40249-020-00734-y. 32883345 10.1186/s40249-020-00734-y PMC7469325

[10] Chui CH, Ko A. Converging humanitarian technology and social work in a public health crisis: a social innovation response to COVID-19 in Hong Kong. Asia Pac J Soc Work Dev. 2021;31(1–2):59–66. 10.1080/02185385.2020.1790412.

[11] Isaiah PM, Sólveig Palmeirim M, Steinmann P. Epidemiology of pediatric schistosomiasis in hard-to-reach areas and populations: a scoping review. Infect Dis Poverty. 2023;12(1):37. 10.1186/s40249-023-01088-x. 37069632 10.1186/s40249-023-01088-x PMC10108517

[12] Munn Z, Moola S, Riitano D, Lisy K. The development of a critical appraisal tool for use in systematic reviews addressing questions of prevalence. IJHPM. 2014;3(3):123–8. 10.15171/ijhpm.2014.71. 25197676 10.15171/ijhpm.2014.71 PMC4154549

[13] Bayram M, Springer S, Garvey CK, Ozdemir V. COVID-19 digital health innovation policy: a portal to alternative futures in the making. OMICS. 2020;24(8):460–9. 10.1089/omi.2020.0089. 32511054 10.1089/omi.2020.0089

[14] Dahlke J, Bogner K, Becker M, Schlaile MP, Pyka A, Ebersberger B. Crisis-driven innovation and fundamental human needs: a typological framework of rapid-response COVID-19 innovations. Technol Forecast Soc Change. 2021. 10.1016/j.techfore.2021.120799. 36540548 10.1016/j.techfore.2021.120799 PMC9755532

[15] Okoń-Horodyńska E. Crisis and innovations: are they constructive or destructive? Stud Log Gramm Rhetor. 2021;66(4):425–49. 10.2478/slgr-2021-0024.

[16] Scheidgen K, Gümüsay AA, Günzel-Jensen F, Krlev G, Wolf M. Crises and entrepreneurial opportunities: Digital social innovation in response to physical distancing. JBV Insights. 2021;15:1–9. 10.1016/j.jbvi.2020.e00222

[17] Haldane V, Foo CD, Abdalla SM, Jung AS, Tan M, Wu SS, et al. Health systems resilience in managing the COVID-19 pandemic: lessons from 28 countries. Nat Med. 2021;27(6):964–80. 10.1038/s41591-021-01381-y. 34002090 10.1038/s41591-021-01381-y

[18] Crawford A, Serhal E. Digital health equity and COVID-19: the innovation curve cannot reinforce the social gradient of health. J Med Internet Res. 2020. 10.2196/19361. 32452816 10.2196/19361 PMC7268667

[19] Romani G, Dal Mas F, Massaro M, Cobianchi L, Modenese M, Barcellini A, et al. Population health strategies to support hospital and intensive care unit resiliency during the COVID-19 pandemic: the Italian experience. Popul Health Manag. 2021;24(2):174–81. 10.1089/pop.2020.0255. 33373536 10.1089/pop.2020.0255

[20] Hengel B, Causer L, Matthews S, Smith K, Andrewartha K, Badman S, et al. A decentralised point-of-care testing model to address inequities in the COVID-19 response. Lancet Infect Dis. 2021;21(7):e183–90. 10.1016/S1473-3099(20)30859-8. 33357517 10.1016/S1473-3099(20)30859-8 PMC7758179

[21] Karim N, Jing L, Austin Lee J, Kharel R, Lubetkin D, Clancy CM, et al. Lessons learned from Rwanda: innovative strategies for prevention and containment of COVID-19. Ann Glob Health. 2021;87:1–9. 10.5334/aogh.3172. 33665145 10.5334/aogh.3172 PMC7908927

[22] Minoi JL, Mohamad FS, Arnab S, Hock ELP. Nudge theory and social innovation: an analysis of citizen and government initiatives during COVID-19 outbreak in Malaysia. In: 2020 IEEE 8th R10 Humanitarian Technology Conference (R10-HTC). Kuching, Malaysia: IEEE; 2020. p. 1–6. 10.1109/R10-HTC49770.2020.9357050

[23] Xinghuan W, Brugère-Picoux J. The COVID-19 battle at CHU Zhongnan and Leishenshan hospital: a summary of the global mobilization in China and reflections on the Wuhan experience. Bull Acad Natl Med. 2021. 10.1016/j.banm.2021.05.007.(InFrench). 34092794 10.1016/j.banm.2021.05.007 PMC8163565

[24] Widhiyoga G, Ikawati N. Health system resilience and community participation amidst the COVID-19 pandemic: a case study of SONJO (Sambatan Jogja) in the Special Region of Yogyakarta, Indonesia. JSP. 2022;26(1):17–32. 10.22146/jsp.68825

[25] Castro-Arroyave DM, Duque-Paz LF. Documentary research on social innovation in health in Latin America. Infect Dis Poverty. 2020;9(1):41. 10.1186/s40249-020-00659-6. 32321575 10.1186/s40249-020-00659-6 PMC7175528

[26] Sseviiri H, Alencar A, Kisira Y. Urban refugees’ digital experiences and social connections during COVID-19 response in Kampala, Uganda. Media Commun. 2022;10(2):276–86. 10.17645/mac.v10i2.5169

[27] Haussig JM, Severi E, Baum JH, Vanlerberghe V, Laiseca A, Defrance L, et al. The European medical corps: first public health team mission and future perspectives. Euro Surveill. 2017. 10.2807/1560-7917.ES.2017.22.37.30613. 28933343 10.2807/1560-7917.ES.2017.22.37.30613 PMC5607656

[28] Cipolla C. Designing with communities of place: the experience of a DESIS Lab during COVID-19 and beyond. SDRJ. 2020;13(3):669–84. 10.4013/sdrj.2020.133.29.

[29] Ha BTT, Ngoc Quang L, Quoc Thanh P, Duc DM, Mirzoev T, Bui TMA. Community engagement in the prevention and control of COVID-19: Insights from Vietnam. PLoS ONE. 2021;16(9):e0254432. 10.1371/journal.pone.0254432. 34495962 10.1371/journal.pone.0254432 PMC8425553

[30] Roscigno G, Yuthavong Y, Manderson LH. Innovation and new technologies to tackle infectious diseases of poverty. In: Global Report for Research on Infectious Diseases of Poverty. World Health Organization; 2012. p. 94–117. https://iris.who.int/bitstream/handle/10665/44850/9789241564489_eng.pdf

[31] Dos Santos MOS, Peixinho BC, Cavalcanti AMC, da Silva LGF, da Silva LIM, Lins DOA, et al. Communication strategies adopted by the management of the Brazilian national health system during the COVID-19 pandemic. Interface Commun Health Educ. 2021;25:e200785. 10.1590/interface.200785

[32] Massey PD, Miller A, Saggers S, Durrheim DN, Speare R, Taylor K, et al. Australian Aboriginal and Torres Strait Islander communities and the development of pandemic influenza containment strategies: community voices and community control. Health Policy. 2011;103(2–3):184–90. 10.1016/j.healthpol.2011.07.004. 21868121 10.1016/j.healthpol.2011.07.004

[33] Cordeiro R, Mont’Alvao C, Quaresma M. Citizen data-driven design for pandemic monitoring. SDRJ. 2020;13(3):342–54. 10.4013/sdrj.2020.133.04.

[34] Nurhasanah IS, Medina-García C, Otieno JN, Balcha WG, Paidakaki A, Van den Broeck P, et al. Social innovation in the face of COVID-19 pandemic. 2020. INSIST Cahier 4. www.insist.earth.international network for social innovation, sustainable development and teritory

[35] Afolabi AA, Ilesanmi EB. Community engagement for COVID-19 prevention and control: a systematic review. Public Health Toxicol. 2022;2(2):1–17. 10.18332/pht/149230

[36] Merrill RD, Chabi AIB, McIntyre E, Kouassi JV, Alleby MM, Codja C, et al. An approach to integrate population mobility patterns and sociocultural factors in communicable disease preparedness and response. Humanit Soc Sci Commun. 2021. 10.1057/s41599-020-00704-7. 38617731 PMC11010577

[37] Sharma S, Singh G, Sharma R, Jones P, Kraus S, Dwivedi YK. Digital health innovation: exploring adoption of cOVID-19 digital contact tracing apps. IEEE Trans Eng Manag. 2020. 10.1109/TEM.2020.3019033.

[38] Ben Abdelaziz A, Berkane S, Ben Salem K, Dahdi SA, Mlouki I, Benzarti S, et al. Lessons learned from the fight against COVID-19 in the Great Maghreb. Five lessons for better resilience. Tunis Med. 2020;98(10):657–63 [French] 33479936

[39] Sharafi Farzad FS, Salamzadeh Y, Amran AB, Hafezalkotob A. Social innovation: towards a better life after COVID-19 crisis: what to concentrate on. JEBE. 2020;8(1):89–120.

[40] Monson K, Oluyinka MJ, Negro DR, Hughes N, Maydan D, Iqbal S, et al. Congregational COVID-19 conversations: utilization of medical-religious partnerships during the SARS-CoV-2 pandemic. J Relig Health. 2021;60(4):2353–61. 10.1007/s10943-021-01290-x. 34032973 10.1007/s10943-021-01290-x PMC8144273

[41] Helms YB, Hamdiui N, Eilers R, Hoebe C, Dukers-Muijrers N, van den Kerkhof H, et al. Online respondent-driven detection for enhanced contact tracing of close-contact infectious diseases: benefits and barriers for public health practice. BMC Infect Dis. 2021. 10.1186/s12879-021-06052-4. 33863279 10.1186/s12879-021-06052-4 PMC8051831

[42] Mason C, Barraket J, Friel S, O’Rourke K, Stenta CP. Social innovation for the promotion of health equity. Health Promot Int. 2015;30(suppl_2):ii116–25. 10.1093/heapro/dav076. 26420807 10.1093/heapro/dav076

[43] Srinivas ML, Yang EJ, Shrestha P, Wu D, Peeling RW, Tucker JD. Social innovation in diagnostics: three case studies. Infect Dis Poverty. 2020;9(1):20. 10.1186/s40249-020-0633-6. 32070433 10.1186/s40249-020-0633-6 PMC7029594

[44] Gebken L, Drews P, Schirmer I. Stakeholder and value orientation in digital social innovation: designing a digital donation concept to support homeless neighbors. In: Proceedings of the Annual Hawaii international conference on system sciences. 2021.

[45] Osborne J, Paget J, Giles-Vernick T, Kutalek R, Napier D, Baliatsas C, et al. Community engagement and vulnerability in infectious diseases: a systematic review and qualitative analysis of the literature. Soc Sci Med. 2021. 10.1016/j.socscimed.2021.114246. 34311391 10.1016/j.socscimed.2021.114246

[46] Tambo E, Djuikoue IC, Tazemda GK, Fotsing MF, Zhou X-N. Early stage risk communication and community engagement (RCCE) strategies and measures against the coronavirus disease 2019 (COVID-19) pandemic crisis. Glob Health J. 2021;5(1):44–50. 10.1016/j.glohj.2021.02.009. 33850632 10.1016/j.glohj.2021.02.009 PMC8032327

[47] Alhassan FM, AlDossary SA. The Saudi Ministry of Health’s twitter communication strategies and public engagement during the COVID-19 pandemic: Content analysis study. JMIR Public Health Surveill. 2021;7(7):e27942. 10.2196/27942. 34117860 10.2196/27942 PMC8276783

[48] Júnior JPB, Morais MB. Community participation in the fight against COVID-19: Between utilitarianism and social justice. Cad Saude Publica. 2020. 10.1590/0102-311x00151620. (Article in English, Portuguese). 10.1590/0102-311x00151620 32756764

[49] Souza CTV, Santana CS, Ferreira P, Nunes JA, Teixeira MLB, Gouvêa M. Caring in the age of COVID-19: lessons from science and society. Cad Saude Publica. 2020;36(6):e00115020. 10.1590/0102-311X00115020. 32609168 10.1590/0102-311X00115020

[50] Patten CA, Balls-Berry JJE, Cohen EL, Brockman TA, Valdez Soto M, West IW, et al. Feasibility of a virtual Facebook community platform for engagement on health research. J Clin Transl Sci. 2021;5(1):e85. 10.1017/cts.2021.12. 34007468 10.1017/cts.2021.12 PMC8111695

[51] Tan CE, Lau SP, Wong SM, Bala P. Innovative use of TPOA telecentres for COVID-19 awareness among the Orang Asli Communities. In: IEEE Region 10 Humanitarian technology conference, R10-HTC; 2020. 10.1109/R10-HTC49770.2020.9357028

[52] Vatan Khah S, Choopani A, Arkian S. How can community engagement help the health system in controlling the COVID-19 pandemic in rural areas? HEHP. 2022;10(2):347–52.

[53] Moscibrodzki P, Ahumuza E, Li J, Sun X, Tao Y, Van Niekerk L, et al. Social innovation in health, community engagement, financing and outcomes: qualitative analysis from the social innovation in health initiative. BMJ Innov. 2022. 10.1136/bmjinnov-2021-000902.

[54] Gilmore B, Ndejjo R, Tchetchia A, de Claro V, Mago E, Lopes C, et al. Community engagement for COVID-19 prevention and control: a rapid evidence synthesis. BMJ Glob Health. 2020;5(10):e003188. 10.1136/bmjgh-2020-003188. 33051285 10.1136/bmjgh-2020-003188 PMC7554411

[55] Frimpong SO, Paintsil E. Community engagement in Ebola outbreaks in sub-Saharan Africa and implications for COVID-19 control: A scoping review. Int J Infect Dis. 2022;126:182–92. 10.1016/j.ijid.2022.11.032. 36462575 10.1016/j.ijid.2022.11.032

[56] Currie WL, Seddon J. Social innovation in public health: can mobile technology make a difference? ISM. 2014;31(3):187–99. 10.1080/10580530.2014.923263.

