# Supplementary material for: Role of social innovations in health in the prevention and control of infectious diseases: a scoping review
Source: Infect Dis Poverty. 2024 Nov 20;13:87. doi: 10.1186/s40249-024-01253-w (PMC11577845; doi:10.1186/s40249-024-01253-w)
Supplement: Supplementary file 1 — Additional file 1. [file 40249_2024_1253_MOESM1_ESM.docx]

**Supplementary 1**

**Search strategy in selected databases**

| **result** | **Strategy search** | **database** |
| --- | --- | --- |
| 744 | (("COVID-19"[Text Word] OR "SARS-CoV-2"[Text Word] OR "SARS CoV 2 Virus"[Text Word] OR "COVID-19 Virus"[Text Word] OR "Novel Coronavirus"[Text Word] OR "Coronavirus"[Text Word] OR "communicable disease control*"[Text Word] OR "Communicable Disease"[Text Word]) AND ("Social Innovation"[Text Word] OR "health innovation"[Text Word] OR "Community Engagement"[Text Word] OR "community participation*"[Text Word])) AND (2010/1/1:2022/12/30[pdat]) | PubMed |
| 790 | **( TITLE-ABS-KEY ( *"Social Innovation"* )  OR  TITLE-ABS-KEY ( *"health innovation"* )  OR  TITLE-ABS-KEY ( *"Community Engagement"* )  OR  TITLE-ABS-KEY ( *"Community Participation*"* ) )  AND  ( TITLE-ABS-KEY ( *"SARS CoV 2 Virus"* )  OR  TITLE-ABS-KEY ( *"COVID-19 Virus"* )  OR  TITLE-ABS-KEY ( *"Novel Coronavirus"* )  OR  TITLE-ABS-KEY ( *coronavirus* )  OR  TITLE-ABS-KEY ( *"Communicable Disease Control*"* )  OR  TITLE-ABS-KEY ( *"Communicable Disease"* ) )  AND  ( LIMIT-TO ( PUBYEAR ,  *2022* )  OR  LIMIT-TO ( PUBYEAR ,  *2021* )  OR  LIMIT-TO ( PUBYEAR ,  *2020* )  OR  LIMIT-TO ( PUBYEAR ,  *2019* )  OR  LIMIT-TO ( PUBYEAR ,  *2018* )  OR  LIMIT-TO ( PUBYEAR ,  *2017* )  OR  LIMIT-TO ( PUBYEAR ,  *2016* )  OR  LIMIT-TO ( PUBYEAR ,  *2015* )  OR  LIMIT-TO ( PUBYEAR ,  *2014* )  OR  LIMIT-TO ( PUBYEAR ,  *2013* )  OR  LIMIT-TO ( PUBYEAR ,  *2012* )  OR  LIMIT-TO ( PUBYEAR ,  *2011* )  OR  LIMIT-TO ( PUBYEAR ,  *2010* ) )** | Scopus |
| 715 | TS=("Social Innovation" OR "health innovation" OR "Community Engagement" OR "Community Participation*") AND TS=("COVID-19" OR "SARS-CoV-2" OR "SARS CoV 2 Virus" OR "COVID-19 Virus" OR "Novel Coronavirus" OR Coronavirus OR "Communicable Disease Control*" OR "Communicable Disease") **Refined by:** **PUBLICATION YEARS:** ( 2022 OR 2021 OR 2016 OR 2012 OR 2020 OR 2015 OR 2011 OR 2019 OR 2013 OR 2010 OR 2018 ) | web of science |
| 2249 | Total results in three database | |
| 26 | Additional source(snowball searching) | |
