# Supplementary material for: Role of social innovations in health in the prevention and control of infectious diseases: a scoping review
Source: Infect Dis Poverty. 2024 Nov 20;13:87. doi: 10.1186/s40249-024-01253-w (PMC11577845; doi:10.1186/s40249-024-01253-w)
Supplement: Supplementary file 2 — Additional file 2. [file 40249_2024_1253_MOESM2_ESM.docx]

**Supplementary 2. : Data collection form- Descriptive characteristics, extracting components and summarizing the results of selected studies for literature review**

| N. Article | title | First Author | Year / journal | Study type | The components of social innovation in the control and management of epidemics | Selected results |
| --- | --- | --- | --- | --- | --- | --- |
| 1 | Contemporary design in quarantine: A critical review of design responses to Covid-19 crisis. | Moura M | 2020/ Strategic Design Research Journal | Review |  |  |
| 2 | Assessing the effectiveness of Social and Political Innovations in the Development of Interaction between the Authorities and the Population during COVID-19: The Implication of Open Innovation. | Kranzeeva E | 2021/ The Implication of Open Innovation | Qualitative |  |  |
| 3 | The application of social innovation in healthcare: a scoping review | van Niekerk L | 2021/ Infectious diseases of poverty | Scoping review |  |  |
| 4 | Fostering social innovation and building adaptive capacity for dengue control in Cambodia: a case study | Echaubard P | 2020/ Infectious diseases of poverty | Case study |  |  |
| 5 | Converging humanitarian technology and social work in a public health crisis: a social innovation response to COVID-19 in Hong Kong | Chui CHK | 2021/ Asia Pacific Journal of Social Work and Development | Qualitative |  |  |
| 6 | COVID-19 Digital Health Innovation Policy: A Portal to Alternative Futures in the Making | Bayram M | 2020/ Omics-a Journal of Integrative Biology | Review |  |  |
| 7 | Crisis-driven innovation and fundamental human needs: A typological framework of rapid-response COVID-19 innovations. | Dahlke J | 2021/ Technological Forecasting and Social Change | Qualitative |  |  |
| 8 | Crisis and Innovations: Are they Constructive or Destructive? | Okoń-Horodyńska E | 2021/ Studies in Logic, Grammar and Rhetoric | Review |  |  |
| 9 | Crises and entrepreneurial opportunities: Digital social innovation in response to physical distancing | Scheidgen K | 2021/ Journal of Business Venturing Insights | Qualitative |  |  |
| 10 | Health systems resilience in managing the COVID-19 pandemic: lessons from 28 countries | Haldane V | 2021/ Nature medicine | Review |  |  |
| 11 | Digital health equity and COVID-19: The innovation curve cannot reinforce the social gradient of health | Crawford A | 2020/ Journal of medical Internet research | Review |  |  |
| 12 | Population Health Strategies to Support Hospital and Intensive Care Unit Resiliency During the COVID-19 Pandemic: The Italian Experience | Romani G | 2021/ Population health management | Review |  |  |
| 13 | A decentralised point-of-care testing model to address inequities in the COVID-19 response | Hengel B | 2021/ The Lancet Infectious Diseases | opinion |  |  |
| 14 | Lessons learned from rwanda: Innovative strategies for prevention and containment of COVID-19 | Karim N | 2021/ Annals of global health | opinion |  |  |
| 15 | Nudge Theory and Social Innovation: An analysis of citizen and government initiatives during Covid-19 outbreak in Malaysia | Minoi JL | 2020/ IEEE Region 10 Humanitarian Technology Conference, R10-HTC | Review |  |  |
| 16 | The COVID-19 battle at CHU Zhongnan and Leishenshan hospital: a summary of the global mobilization in China and reflections on the Wuhan experience | Xinghuan W | 2021/ Bulletin de l'Academie nationale de medecine | Review |  |  |
| 17 | Health System Resilience and Community Participation amidst the Covid 19 Pandemic: A Case Study of SONJO (Sambatan Jogja) in the Special Region of Yogyakarta, Indonesia | Widhiyoga G | 2022/. Jurnal Ilmu Sosial dan Ilmu Politik | Qualitative |  |  |
| 18 | Documentary research on social innovation in health in Latin America | Castro-Arroyave DM | 2020/ Infectious diseases of poverty | Scoping review |  |  |
| 19 | Urban Refugees’ Digital Experiences and Social Connections During Covid‐19 Response in Kampala, Uganda | Sseviiri H | 2022/ Media and Communication | Qualitative |  |  |
| 20 | The European medical corps: First public health team mission and future perspectives | Haussig JM | 2022/Media and Communication | Qualitative |  |  |
| 21 | Designing with communities of place: The experience of a DESIS Lab during COVID-19 and beyond | Cipolla C | 2020/ Strategic Design Research Journal | Case report |  |  |
| 22 | Community engagement in the prevention and control of COVID-19: Insights from Vietnam | Ha BTT | 2021/ PloS one | Quantitative |  |  |
| 23 | Innovation and new technologies to tackle infectious diseases of poverty. | Roscigno G | 2012/ Global Report for Research on Infectious Diseases of Poverty: World Health Organization | Report-qualitative |  |  |
| 24 | Communication strategies adopted by the management of the brazilian national health system during the covid-19 pandemic. | Dos Santos | 2021/ Interface: Communication, Health, Education | Qualitative |  |  |
| 25 | Australian Aboriginal and Torres Strait Islander communities and the development of pandemic influenza containment strategies: community voices and community control. | Massey PD | 2011/ Health policy | Qualitative |  |  |
| 26 | Citizen data-driven design for pandemic monitoring | Cordeiro R | 2020/Strategic Design Research Journal | Qualitative |  |  |
| 27 | Social Innovation in the Face of COVID-19 Pandemic | Nurhasanah IS | 2020/ | Report-qualitative |  |  |
| 28 | Community engagement for COVID-19 prevention and control: A systematic review | Afolabi AA | 2022/ J Public Health Toxicology | Systematic review |  |  |
| 29 | An approach to integrate population mobility patterns and sociocultural factors in communicable disease preparedness and response | Merrill RD | 2021/Humanities and Social Sciences Communications | Qualitative |  |  |
| 30 | Digital Health Innovation: Exploring Adoption of COVID-19 Digital Contact Tracing Apps. | Sharma S | 2020/IEEE Transactions on Engineering Management | Quantitative |  |  |
| 31 | Lessons learned from the fight against COVID-19 in the Great Maghreb.Five lessons for better resilience. | Ben Abdelaziz A | 2020/ La Tunisie medicale | Qualitative |  |  |
| 32 | Social Innovation: Towards a better life after COVID-19 crisis: What to concentrate on. | Sharafi Farzad F | 2020/ Journal of Entrepreneurship, Business and Economics | Qualitative |  |  |
| 33 | Congregational COVID-19 Conversations: Utilization of Medical-Religious Partnerships During the SARS-CoV-2 Pandemic | Monson K | 2021/Journal of religion and health | Review |  |  |
| 34 | Online respondent-driven detection for enhanced contact tracing of close-contact infectious diseases: benefits and barriers for public health practice. | Helms YB | 2021/ BMC Infectious Diseases | mixed methods |  |  |
| 35 | Social innovation for the promotion of health equity. | Mason C | 2015/ Health promotion international | Review |  |  |
| 36 | Social innovation in diagnostics: three case studies | Srinivas ML | 2020/ Infectious diseases of poverty | Review |  |  |
| 37 | Stakeholder and value orientation in digital social innovation: Designing a digital donation concept to support homeless neighbors. | Gebken L | 2021/Proceedings of the Annual Hawaii International Conference on System Sciences; 2021 | Case report |  |  |
| 38 | Community engagement and vulnerability in infectious diseases: A systematic review and qualitative analysis of the literature. | Osborne J | 2021/ Social Science and Medicine | Qualitative |  |  |
| 39 | Early stage risk communication and community engagement (RCCE) strategies and measures against the coronavirus disease 2019 (COVID-19) pandemic crisis | Tambo E | 2021/ Global Health Journa | Review |  |  |
| 40 | The Saudi Ministry of Health's Twitter Communication Strategies and Public Engagement During the COVID-19 Pandemic: Content Analysis Study | Alhassan FM | 2021/ JMIR public health and surveillance | Qualitative |  |  |
| 41 | Community participation in the fight against COVID-19: Between utilitarianism and social justice. | Júnior JPB | 2020/ Cadernos de saude publica | Review |  |  |
| 42 | Caring in the age of COVID-19: lessons from science and society | Souza CTV | 2020/ Cadernos de saude publica | Qualitative |  |  |
| 43 | Feasibility of a virtual Facebook community platform for engagement on health research | Patten CA | 2021/ Journal of clinical and translational science | Case study |  |  |
| 44 | Congregational COVID-19 Conversations: Utilization of Medical-Religious Partnerships During the SARS-CoV-2 Pandemic. | Monson K | 2021/ Journal of religion and health | Qualitative |  |  |
| 45 | Innovative Use of TPOA Telecentres for Covid-19 Awareness among the Orang Asli Communities. | Tan CE | 2020/ IEEE Region 10 Humanitarian Technology Conference, R10-HTC; 2020 | Case report |  |  |
| 46 | How Can Community Engagement Help the Health System in Controlling the COVID-19 Pandemic in Rural Areas? | Vatan Khah S | 2022/ Promotion H. | Qualitative |  |  |
| 47 | Social innovation in health, community engagement, financing and outcomes: qualitative analysis from the social innovation in health initiative | Moscibrodzki p | 2022/ BMJ Innovations | Qualitative |  |  |
| 48 | Community engagement for COVID-19 prevention and control: a rapid evidence synthesis | Gilmore B | 2020/ BMJ global health | Qualitative |  |  |
| 49 | Community engagement in Ebola outbreaks in sub-Saharan Africa and implications for COVID-19 control: A scoping review. | Frimpong SO | 2022/ International journal of infectious diseases | Scoping review |  |  |
| 50 | Social innovation in public health: can mobile technology make a difference? | Currie WL | 2014/ Seddon JJJISM. | Quantitatve |  |  |
