# Supplementary material for: Role of social innovations in health in the prevention and control of infectious diseases: a scoping review
Source: Infect Dis Poverty. 2024 Nov 20;13:87. doi: 10.1186/s40249-024-01253-w (PMC11577845; doi:10.1186/s40249-024-01253-w)
Supplement: Supplementary file 3 — Additional file 3. [file 40249_2024_1253_MOESM3_ESM.docx]

**Supplementary 3:** Quality Assessment Report of Articles Included in the scoping Review

the quality of the selected articles was assessed using the Joanna Briggs Institute Prevalence Critical Appraisal Tool(1). Each article was scored using 10 quality control components suggested by this tool, so that one point was considered for each item. Finally, the total scores were categorized based on (7-10) high quality, (4-6) medium and (3-0) low quality(2). The tool assessed each article on the questions following:

1. Sample representative of the target population
2. Study participants recruited in an appropriate way
3. Sample size adequate
4. Study subjects and setting described in detail
5. Data analysis was conducted with enough coverage of the identified sample
6. Validity of methods used to identify the condition of interest
7. Condition measured in a standard, reliable way for all participants
8. Appropriate statistical analysis
9. Adequacy of response rate; were low response rates managed?
10. Sub-population of interest identified using objective criteria

| Author(s) and Year | Was the sample representative of the target population? | Were study participants recruited in an appropriate way? | Was the sample size adequate? | Were the study subjects and setting described in detail? | Was data analysis conducted with sufficient coverage of the identified sample? | Were valid methods used for the identification of the condition? | Was the condition measured in a standard, reliable way for all participants? | Was there appropriate statistical analysis? | Was the response rate adequate, and if not, was the low response rate managed appropriately? | Were target subpopulations identified using objective criteria? | Quality score |
| --- | --- | --- | --- | --- | --- | --- | --- | --- | --- | --- | --- |
| Moura M. et al.,2020 | Yes | Yes | Yes | Yes | Yes | Yes | Yes | Yes | NA | Yes | 9 |
| Kranzeeva E et al.,2021 | Yes | Yes | Yes | Yes | Yes | Yes | Yes | Yes | NA | Yes | 9 |
| van Niekerk L et al.,2021 | Yes | Yes | Yes | Yes | Yes | Yes | Yes | Yes | NA | Yes | 9 |
| Echaubard P et al.,2020 | Yes | Yes | Yes | Yes | Yes | Not specified | Yes | Yes | NA | Yes | 8 |
| Chui CHK et al.,2021 | Yes | Not specified | Not specified | Yes | Yes | Not specified | Yes | Yes | NA | Yes | 6 |
| Bayram M et al.,2020 | Yes | Not specified | Not specified | Yes | Yes | Yes | Yes | Yes | NA | Yes | 7 |
| Dahlke J et al.,2021 | Yes | Yes | Yes | Yes | Yes | Yes | Yes | Yes | NA | Yes | 9 |
| Okoń-Horodyńska E et al.,2021 | Yes | Not specified | Not specified | Yes | Yes | Not specified | NA | Yes | NA | Yes | 5 |
| Scheidgen K et al.,2021 | Yes | Yes | Yes | Yes | Yes | Yes | Yes | Yes | NA | Yes | 9 |
| Haldane V et al., 2021 | Yes | Yes | Yes | Yes | Yes | Yes | Yes | Yes | NA | Yes | 9 |
| Crawford A et al., 2020 | No | Yes | Yes | Yes | Not specified | Not specified | NA | Yes | NA | Yes | 5 |
| Romani G et al., 2021 | Yes | Yes | Yes | Yes | Yes | Yes | Yes | Yes | NA | Yes | 9 |
| Hengel B et al., 2021 | Yes | Yes | Yes | Yes | Not specified | Yes | Yes | Yes | NA | Yes | 8 |
| Karim N et al., 2021 | Yes | Yes | Not specified | Yes | Yes | Yes | Yes | Yes | NA | Yes | 8 |
| Minoi JL et al.,2020 | No | Yes | No | Yes | Yes | Not specified | Yes | NA | NA | Yes | 5 |
| Xinghuan W et al., 2021 | Yes | Not specified | Not specified | Yes | Yes | Yes | Yes | Yes | NA | Yes | 7 |
| Widhiyoga G et al.,2022 | Yes | Yes | Yes | Yes | Yes | Yes | Yes | Yes | NA | Yes | 9 |
| Castro-Arroyave DM et al.,2020 | Yes | Yes | Yes | Yes | Yes | Yes | Yes | Yes | NA | Yes | 9 |
| Sseviiri H et al.,2022 | Yes | Yes | Yes | Yes | Yes | Yes | Yes | Yes | NA | Yes | 9 |
| Haussig JM et al.,2022 | Yes | Not specified | Not specified | Yes | Yes | Yes | Yes | Yes | NA | Yes | 7 |
| Author(s) and Year | Was the sample representative of the target population? | Were study participants recruited in an appropriate way? | Was the sample size adequate? | Were the study subjects and setting described in detail? | Was data analysis conducted with sufficient coverage of the identified sample? | Were valid methods used for the identification of the condition? | Was the condition measured in a standard, reliable way for all participants? | Was there appropriate statistical analysis? | Was the response rate adequate, and if not, was the low response rate managed appropriately? | Were target subpopulations identified using objective criteria? | Quality score |
| Cipolla C et al.,2020 | Yes | Yes | Yes | Yes | Yes | Yes | Yes | Yes | NA | Yes | 9 |
| Ha BTT et al.,2021 | Yes | Yes | Yes | Yes | Yes | Yes | Yes | Yes | NA | Yes | 9 |
| Roscigno G et al.,2012 | Yes | Not specified | Yes | Yes | Yes | Yes | Yes | Not specified | NA | Yes | 7 |
| Dos Santos et al.,2021 | Yes | Yes | Yes | Yes | Yes | Yes | Yes | Yes | NA | Yes | 9 |
| Massey PD et al.,2011 | Yes | Yes | Yes | Yes | Yes | Yes | Yes | Yes | NA | Yes | 9 |
| Cordeiro R et al.,2020 | Yes | Yes | Yes | Yes | Yes | Yes | Yes | Yes | NA | Yes | 9 |
| Nurhasanah IS et al.,2020 | Yes | Not specified | Yes | Yes | Yes | Yes | Yes | Not specified | NA | Yes | 7 |
| Afolabi AA et al.,2022 | Yes | Yes | Yes | Yes | Yes | Yes | Yes | Yes | NA | Yes | 9 |
| Merrill RD et al.,2021 | Yes | Yes | Yes | Yes | Yes | Yes | Yes | Yes | NA | Yes | 9 |
| Sharma S et al.,2020 | Yes | Yes | Yes | Yes | Yes | Yes | Yes | Yes | NA | Yes | 9 |
| Ben Abdelaziz A et al.,2020 | Yes | Yes | Yes | Yes | Yes | Yes | Yes | Not specified | NA | Yes | 8 |
| Sharafi Farzad F et al.,2020 | Yes | Yes | Yes | Yes | Yes | Yes | Yes | Yes | NA | Yes | 9 |
| Monson K et al.,2021 | Not specified | Yes | Not specified | Yes | Yes | Yes | Yes | Yes | NA | Yes | 7 |
| Helms YB et al.,2021 | Yes | Yes | Yes | Yes | Yes | Yes | Yes | Yes | NA | Yes | 9 |
| Mason C et al.,2015 | Yes | Yes | Yes | Yes | Yes | Yes | Yes | Yes | NA | Yes | 9 |
| Srinivas ML et al.,2020 | Yes | Not specified | Not specified | Yes | Yes | Yes | Yes | Yes | NA | Yes | 7 |
| Gebken L et al.,2021 | Yes | Yes | Yes | Yes | Yes | Yes | Yes | Yes | NA | Yes | 9 |
| Osborne J et al.,2021 | Yes | Yes | Yes | Yes | Yes | Yes | Yes | Yes | NA | Yes | 9 |
| Tambo E et al.,2021 | No | Not specified | Yes | Yes | Yes | Yes | Yes | Yes | NA | Yes | 7 |
| Alhassan FM et al.,2021 | Yes | Yes | Yes | Yes | Yes | Yes | Yes | Yes | NA | Yes | 9 |
| Júnior JPB et al.,2020 | Yes | Not specified | Not specified | Yes | Yes | Yes | Yes | Yes | NA | Yes | 7 |
| Souza CTV et al.,2020 | Yes | Yes | Not specified | Yes | Yes | Yes | Yes | Not specified | NA | Yes | 7 |
| Patten CA et al.,2021 | Yes | Yes | Yes | Yes | Yes | Yes | Yes | Yes | NA | Yes | 9 |
| Monson K et al.,2021 | Yes | Not specified | Not specified | Yes | Yes | Yes | Yes | Yes | NA | Yes | 7 |
| Tan CE et al.,2020 | Yes | Yes | Yes | Yes | Yes | Yes | Yes | Yes | NA | Yes | 9 |
| Vatan Khah S et al.,2022 | Yes | Yes | Yes | Yes | Yes | Yes | Yes | Yes | NA | Yes | 9 |
| Moscibrodzki p et al.,2022 | Yes | Yes | Yes | Yes | Yes | Yes | Yes | Yes | NA | Yes | 9 |
| Gilmore B et al.,2020 | Yes | Yes | Yes | Yes | Yes | Yes | Yes | Yes | NA | Yes | 9 |
| Frimpong SO et al.,2022 | Yes | Yes | Yes | Yes | Yes | Yes | Yes | Yes | NA | Yes | 9 |
| Currie WL et al.,2014 | Yes | Yes | Yes | Yes | Yes | Yes | Yes | Yes | NA | Yes | 9 |

**Reference**

1. Isaiah PM, Sólveig Palmeirim M, Steinmann P. Epidemiology of pediatric schistosomiasis in hard-to-reach areas and populations: a scoping review. Infectious diseases of poverty. 2023;12(1):37.

2. Munn Z, Moola S, Riitano D, Lisy K. The development of a critical appraisal tool for use in systematic reviews addressing questions of prevalence. International journal of health policy and management. 2014;3(3):123-8.
