# Supplementary material for: Role of social innovations in health in the prevention and control of infectious diseases: a scoping review
Source: Infect Dis Poverty. 2024 Nov 20;13:87. doi: 10.1186/s40249-024-01253-w (PMC11577845; doi:10.1186/s40249-024-01253-w)
Supplement: Supplementary file 4 — Additional file 4. [file 40249_2024_1253_MOESM4_ESM.docx]

**Information 50 documents used for analysis and extraction results**

| N | N. references in text article | First Author | title | Type-research | Year / journal |
| --- | --- | --- | --- | --- | --- |
| 1 | 3 | Moura M | Contemporary design in quarantine: A critical review of design responses to Covid-19 crisis. | Review | 2020/ Strategic Design Research Journal |
| 2 | 5 | Kranzeeva E | Assessing the effectiveness of Social and Political Innovations in the Development of Interaction between the Authorities and the Population during COVID-19: The Implication of Open Innovation. | Qualitative | 2021/ The Implication of Open Innovation |
| 3 | 8 | van Niekerk L | The application of social innovation in healthcare: a scoping review | Scoping review | 2021/ Infectious diseases of poverty |
| 4 | 9 | Echaubard P | Fostering social innovation and building adaptive capacity for dengue control in Cambodia: a case study | Case study | 2020/ Infectious diseases of poverty |
| 5 | 10 | Chui CHK | Converging humanitarian technology and social work in a public health crisis: a social innovation response to COVID-19 in Hong Kong | Qualitative | 2021/ Asia Pacific Journal of Social Work and Development |
| 6 | 13 | Bayram M | COVID-19 Digital Health Innovation Policy: A Portal to Alternative Futures in the Making | Review | 2020/ Omics-a Journal of Integrative Biology |
| 7 | 14 | Dahlke J | Crisis-driven innovation and fundamental human needs: A typological framework of rapid-response COVID-19 innovations. | Qualitative | 2021/ Technological Forecasting and Social Change |
| 8 | 15 | Okoń-Horodyńska E | Crisis and Innovations: Are they Constructive or Destructive? | Review | 2021/ Studies in Logic, Grammar and Rhetoric |
| 9 | 16 | Scheidgen K | Crises and entrepreneurial opportunities: Digital social innovation in response to physical distancing | Qualitative | 2021/ Journal of Business Venturing Insights |
| 10 | 17 | Haldane V | Health systems resilience in managing the COVID-19 pandemic: lessons from 28 countries | Review | 2021/ Nature medicine |
| 11 | 18 | Crawford A | Digital health equity and COVID-19: The innovation curve cannot reinforce the social gradient of health | Review | 2020/ Journal of medical Internet research |
| 12 | 19 | Romani G | Population Health Strategies to Support Hospital and Intensive Care Unit Resiliency During the COVID-19 Pandemic: The Italian Experience | Review | 2021/ Population health management |
| 13 | 20 | Hengel B | A decentralised point-of-care testing model to address inequities in the COVID-19 response | opinion | 2021/ The Lancet Infectious Diseases |
| 14 | 21 | Karim N | Lessons learned from rwanda: Innovative strategies for prevention and containment of COVID-19 | opinion | 2021/ Annals of global health |
| 15 | 22 | Minoi JL | Nudge Theory and Social Innovation: An analysis of citizen and government initiatives during Covid-19 outbreak in Malaysia | Review | 2020/ IEEE Region 10 Humanitarian Technology Conference, R10-HTC |
| 16 | 23 | Xinghuan W | The COVID-19 battle at CHU Zhongnan and Leishenshan hospital: a summary of the global mobilization in China and reflections on the Wuhan experience | Review | 2021/ Bulletin de l'Academie nationale de medecine |
| 17 | 24 | Widhiyoga G | Health System Resilience and Community Participation amidst the Covid 19 Pandemic: A Case Study of SONJO (Sambatan Jogja) in the Special Region of Yogyakarta, Indonesia | Qualitative | 2022/. Jurnal Ilmu Sosial dan Ilmu Politik |
| 18 | 25 | Castro-Arroyave DM | Documentary research on social innovation in health in Latin America | Scoping review | 2020/ Infectious diseases of poverty |
| 19 | 26 | Sseviiri H | Urban Refugees’ Digital Experiences and Social Connections During Covid‐19 Response in Kampala, Uganda | Qualitative | 2022/ Media and Communication |
| 20 | 27 | Haussig JM | The European medical corps: First public health team mission and future perspectives | Qualitative | 2022/Media and Communication |
| 21 | 28 | Cipolla C | Designing with communities of place: The experience of a DESIS Lab during COVID-19 and beyond | Case report | 2020/ Strategic Design Research Journal |
| 22 | 29 | Ha BTT | Community engagement in the prevention and control of COVID-19: Insights from Vietnam | Quantitative | 2021/ PloS one |
| 23 | 30 | Roscigno G | Innovation and new technologies to tackle infectious diseases of poverty. | Report-qualitative | 2012/ Global Report for Research on Infectious Diseases of Poverty: World Health Organization |
| 24 | 31 | Dos Santos | Communication strategies adopted by the management of the brazilian national health system during the covid-19 pandemic. | Qualitative | 2021/ Interface: Communication, Health, Education |
| 25 | 32 | Massey PD | Australian Aboriginal and Torres Strait Islander communities and the development of pandemic influenza containment strategies: community voices and community control. | Qualitative | 2011/ Health policy |
| 26 | 33 | Cordeiro R | Citizen data-driven design for pandemic monitoring | Qualitative | 2020/Strategic Design Research Journal |
| 27 | 34 | Nurhasanah IS | Social Innovation in the Face of COVID-19 Pandemic | Report-qualitative | 2020/ |
| 28 | 35 | Afolabi AA | Community engagement for COVID-19 prevention and control: A systematic review | Systematic review | 2022/ J Public Health Toxicology |
| 29 | 36 | Merrill RD | An approach to integrate population mobility patterns and sociocultural factors in communicable disease preparedness and response | Qualitative | 2021/Humanities and Social Sciences Communications |
| 30 | 37 | Sharma S | Digital Health Innovation: Exploring Adoption of COVID-19 Digital Contact Tracing Apps. | Quantitative | 2020/IEEE Transactions on Engineering Management |
| 31 | 38 | Ben Abdelaziz A | Lessons learned from the fight against COVID-19 in the Great Maghreb.Five lessons for better resilience. | Qualitative | 2020/ La Tunisie medicale |
| 32 | 39 | Sharafi Farzad F | Social Innovation: Towards a better life after COVID-19 crisis: What to concentrate on. | Qualitative | 2020/ Journal of Entrepreneurship, Business and Economics |
| 33 | 40 | Monson K | Congregational COVID-19 Conversations: Utilization of Medical-Religious Partnerships During the SARS-CoV-2 Pandemic | Review | 2021/Journal of religion and health |
| 34 | 41 | Helms YB | Online respondent-driven detection for enhanced contact tracing of close-contact infectious diseases: benefits and barriers for public health practice. | mixed methods | 2021/ BMC Infectious Diseases |
| 35 | 42 | Mason C | Social innovation for the promotion of health equity. | Review | 2015/ Health promotion international |
| 36 | 43 | Srinivas ML | Social innovation in diagnostics: three case studies | Review | 2020/ Infectious diseases of poverty |
| 37 | 44 | Gebken L | Stakeholder and value orientation in digital social innovation: Designing a digital donation concept to support homeless neighbors. | Case report | 2021/Proceedings of the Annual Hawaii International Conference on System Sciences; 2021 |
| 38 | 45 | Osborne J | Community engagement and vulnerability in infectious diseases: A systematic review and qualitative analysis of the literature. | Qualitative | 2021/ Social Science and Medicine |
| 39 | 46 | Tambo E | Early stage risk communication and community engagement (RCCE) strategies and measures against the coronavirus disease 2019 (COVID-19) pandemic crisis | Review | 2021/ Global Health Journa |
| 40 | 47 | Alhassan FM | The Saudi Ministry of Health's Twitter Communication Strategies and Public Engagement During the COVID-19 Pandemic: Content Analysis Study | Qualitative | 2021/ JMIR public health and surveillance |
| 41 | 48 | Júnior JPB | Community participation in the fight against COVID-19: Between utilitarianism and social justice. | Review | 2020/ Cadernos de saude publica |
| 42 | 49 | Souza CTV | Caring in the age of COVID-19: lessons from science and society | Qualitative | 2020/ Cadernos de saude publica |
| 43 | 50 | Patten CA | Feasibility of a virtual Facebook community platform for engagement on health research | Case study | 2021/ Journal of clinical and translational science |
| 44 | 51 | Monson K | Congregational COVID-19 Conversations: Utilization of Medical-Religious Partnerships During the SARS-CoV-2 Pandemic. | Qualitative | 2021/ Journal of religion and health |
| 45 | 52 | Tan CE | Innovative Use of TPOA Telecentres for Covid-19 Awareness among the Orang Asli Communities. | Case report | 2020/ IEEE Region 10 Humanitarian Technology Conference, R10-HTC; 2020 |
| 46 | 53 | Vatan Khah S | How Can Community Engagement Help the Health System in Controlling the COVID-19 Pandemic in Rural Areas? | Qualitative | 2022/ Promotion H. |
| 47 | 54 | Moscibrodzki p | Social innovation in health, community engagement, financing and outcomes: qualitative analysis from the social innovation in health initiative | Qualitative | 2022/ BMJ Innovations |
| 48 | 55 | Gilmore B | Community engagement for COVID-19 prevention and control: a rapid evidence synthesis | Qualitative | 2020/ BMJ global health |
| 49 | 56 | Frimpong SO | Community engagement in Ebola outbreaks in sub-Saharan Africa and implications for COVID-19 control: A scoping review. | Scoping review | 2022/ International journal of infectious diseases |
| 50 | 57 | Currie WL | Social innovation in public health: can mobile technology make a difference? | Quantitatve | 2014/ Seddon JJJISM. |
